# Patient Reported and Structural Outcomes of Knee Joint Distraction versus High Tibial Osteotomy and Total Knee Arthroplasty: A Meta-Analysis

**DOI:** 10.5704/MOJ.2511.010

**Published:** 2025-11

**Authors:** D Tan, BS Angeles, CL Braganza

**Affiliations:** Department of Orthopaedics, University of Santo Tomas Hospital, Manila, Philippines

**Keywords:** knee joint distraction (KJD), high tibial osteotomy (HTOTKA), severe knee osteoarthritis

## Abstract

**Introduction:**

Knee osteoarthritis (OA) is a degenerative joint condition with significant global prevalence, often resulting from inflammatory joint processes, trauma, occupational stress, and obesity. While total knee arthroplasty (TKA) effectively alleviates pain and restores function, its limited lifespan and increased revision risk in younger patients necessitate alternative joint-preserving strategies. Emerging evidence highlights the potential of approaches such as Knee Joint Distraction (KJD), High Tibial Osteotomy (HTO), platelet-rich plasma therapy, and radiofrequency ablation in managing knee OA. These non-invasive and joint-preserving interventions have demonstrated efficacy in reducing OA-related pain and improving patient outcomes.

**Material and Methods:**

This study evaluated four comparative studies focusing on KJD vs HTO and TKA in the treatment of severe knee OA. Patient-reported outcomes were assessed using validated tools, including the Western Ontario and McMaster Universities Osteoarthritis Index (WOMAC), Knee injury and Osteoarthritis Outcome Score (KOOS), Intermittent and Constant Osteoarthritis Pain (ICOAP) score, Visual Analog Scale (VAS) for pain, EuroQol-5 Dimensions (EQ-5D), and Short Form-36 (SF-36). Structural outcomes were quantified via Joint Space Width (JSW), an indicator of cartilage preservation. Data were analysed using Review Manager (RevMan) version 5, with Cochrane’s Q test applied to evaluate heterogeneity. Results were summarised using Forest plots, and statistical significance was set at p < 0.05.

**Results:**

Statistical analysis revealed significant differences between KJD vs. HTO and TKA across all measured outcomes. HTO and TKA demonstrated superior improvements in WOMAC, KOOS, ICOAP, VAS, EQ-5D, SF-36, and JSW. Despite these statistically significant differences, the mean values were comparable, suggesting KJD’s non-inferiority as a joint-preserving alternative. The efficacy of non-invasive modalities in alleviating knee OA symptoms further strengthens the argument for exploring less invasive, cost-effective options for managing this condition.

**Conclusion:**

Knee Joint Distraction emerges as a promising joint-preserving intervention, offering comparable pain relief and functional improvement to HTO and TKA in the management of severe knee OA. While HTO and TKA showed marginally superior outcomes, KJD remains a viable alternative for younger patients or those seeking to delay TKA. Incorporating adjunctive treatments such as platelet-rich plasma therapy or radiofrequency ablation may further enhance outcomes, paving the way for multimodal and individualised approaches to knee OA management.

## Introduction

Knee osteoarthritis (KOA) is one of the most prevalent forms of osteoarthritis and is a leading cause of disability worldwide. This condition represents a major global health burden, with its incidence expected to rise in the coming decades^1^. For patients with end-stage knee osteoarthritis, total knee arthroplasty (TKA) is the standard surgical intervention, widely regarded as an effective and safe option^2,3^. However, in younger and more active patients, TKA is associated with an increased risk of failure and the need for revision surgery^4^. These limitations highlight the importance of exploring alternative joint-preserving procedures that may delay or reduce the need for TKA.

Among joint-preserving procedures, High Tibial Osteotomy (HTO) and Knee Joint Distraction (KJD) are notable options. HTO is a well-established approach for patients with unicompartmental knee osteoarthritis, offering good long-term survival and improvements in patient-reported outcomes^5-9^. On the other hand, KJD is a relatively novel technique that promotes cartilage repair and offers long-term clinical benefits for patients with unilateral or bicompartmental tibiofemoral osteoarthritis and minimal malalignment^10-15^. Both procedures aim to preserve the native joint tissue, making them appealing alternatives to TKA in appropriately selected patients.

While these joint-preserving approaches are promising, TKA remains the gold standard for managing advanced knee osteoarthritis^2^. As such, comparing the outcomes of joint-preserving procedures like HTO and KJD with TKA is crucial to better understand their efficacy and utility in clinical practice. Despite the growing interest in these procedures, there remains a paucity of comprehensive comparative studies and meta-analyses exploring their patient-reported and structural outcomes^16,17^.

This study aims to address this gap by comparing Knee Joint Distraction, High Tibial Osteotomy and Total Knee Arthroplasty in patients with severe knee osteoarthritis. The comparison focuses on patient-reported outcomes, including the Western Ontario and McMaster Universities Arthritis Index (WOMAC), Knee Injury and Osteoarthritis Outcome Score (KOOS), Intermittent and Constant Pain Score (ICOAP), Visual Analog Scale (VAS), Euro Quality of Life 5D Score (EQ5D), and Short Form Health Survey (SF36). Structural changes are assessed using the mean Joint Space Width (JSW). By conducting this comparative assessment, this research seeks to provide valuable insights into the outcomes of these procedures and contribute to the limited body of literature on joint-preserving treatments for knee osteoarthritis.

Inclusion of a Filipino study that have explored these areas was not made. To date, no current Filipino authored study published internationally or locally could be used in the actual review.

## Materials and Methods

The principal objective of this study was to provide a comparative assessment between Knee Joint Distraction (KJD), High Tibial Osteotomy (HTO) and Total Knee Arthroplasty (TKA) in patients with severe knee osteoarthritis in terms of patient-reported outcomes and structural changes.

This study utilised a meta-analysis research design. Relevant studies were identified by systematically searching multiple databases and synthesising the findings to compare the outcomes of KJD, HTO and TKA.

The systematic search was conducted across several electronic databases, including PubMed, BioMed Central (BMC), Ebscohost, the Cochrane Library, and Google Scholar. The search spanned from 2015 to 2023. The following keywords and Boolean operators were employed: “knee joint distraction” AND “high tibial osteotomy” AND “total knee arthroplasty” AND “severe knee osteoarthritis” AND “outcomes of joint preservation surgeries”.

Filters were applied to include randomised controlled trials (RCTs) and full-text articles published in English. Backward citation tracking was performed by reviewing the references of the selected studies to identify additional relevant articles. In addition to the above keywords, Boolean operators like AND/OR were employed to refine the search. Filters were applied to include randomised controlled trials (RCTs) and full-text articles published in English. Studies not published in English or lacking full-text availability were excluded. Furthermore, backward citation tracking was performed by reviewing the references of the selected studies to identify additional relevant articles. The number of articles retrieved from each database was as follows: PubMed (5), Cochrane Library (2), Google Scholar (2).

The total number of records retrieved was 9, which were further processed through screening and inclusion/exclusion criteria. The study was conducted throughout 2023 including database search, screening, data extraction, analysis, and reporting.

Randomised controlled trials comparing Knee Joint Distraction (KJD), High Tibial Osteotomy (HTO) and Total Knee Arthroplasty (TKA). Studies assessing severe knee osteoarthritis in patients aged ≥18 years^18^. Articles reporting patient-reported outcomes (e.g., WOMAC, KOOS, ICOAP, VAS, EQ5D, SF36) and/or structural changes (mean Joint Space Width) and Full-text studies published in English.

Non-randomised studies, observational studies, reviews, or case reports, Studies without full-text availability, Articles involving knee conditions other than osteoarthritis (e.g., post-traumatic arthritis) and Studies with insufficient data or lacking relevant outcome measures.

Studies retrieved from database searches were saved as PDF files. These files were manually categorised using Microsoft Excel to document study characteristics, including study design, year and country of publication, sample size, and outcomes measured. Research studies meeting the selection criteria were stored securely on a password-protected hard drive accessible only to the main author and co-proponent.

The main author and co-proponent independently reviewed the selected full-text articles to extract relevant data. Data collected included study design, publication year, patient demographics, number of participants for each surgical approach, reported outcomes, and dropouts. The extracted data were cross-verified, and discrepancies resolved through discussion until consensus was achieved.

The Review Manager (RevMan) version 5 software was used to evaluate the risk of bias in each included study. Studies were assessed based on criteria such as random sequence generation, allocation concealment, blinding, handling of incomplete outcome data, selective reporting, and other biases. Each criterion was rated as “low risk,” “high risk,” or “unclear risk”. Risk of bias assessments were conducted independently by the main author and co-proponent, and any disagreements were resolved through discussion.

**Fig. 1:**
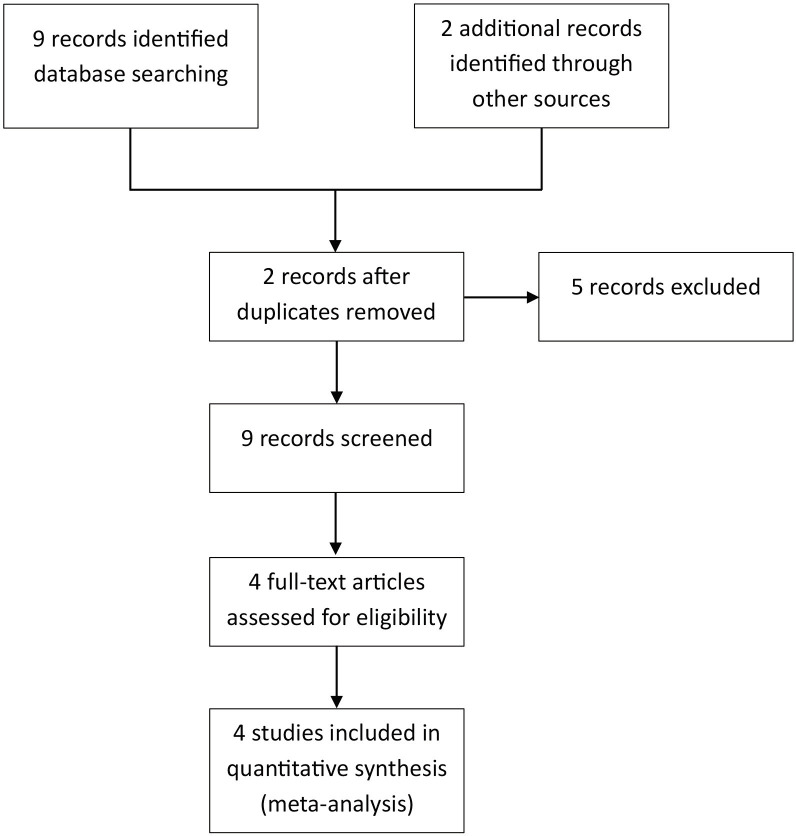
Prisma flowchart of the study.

Based on Fig. 1 Prisma flowchart outlines the study selection process. The search initially retrieved nine records through database searches (PubMed: five, Cochrane Library: two, Google Scholar: two) and an additional two studies through backward citation tracking. After removing two duplicates, nine studies were screened. Five studies were excluded for the following reasons: irrelevant population (n=2), insufficient outcome data (n=2), and inappropriate study design (n=1). Four studies met the inclusion criteria and were included in the final analysis.

On Table I^19-22^, consistent with findings by van der Woude *et al*^20^ the included studies were all conducted in the Netherlands and utilised a randomised controlled trial design. Follow-up periods ranged from baseline to 12 and 24 months. The study by Wiegant *et al*^22^ had the largest sample size, with 67 patients at baseline and 100 at follow-up. The mean age of all participants across studies was 53 years.

**Table I:** Summary of study characteristics.

Study	Year	Country	SD	FU (months)	No. at FU	Sample size	Mean age
Jansen *et al*^19^	2019	Netherlands	RCT	0, 12, 24	60	60	52.5±6.8
van Der Woude *et al*^20^	2017	Netherlands	RCT	0, 3, 6, 9, 12	56	56	55.0±1.4
van Der Woude *et al*^21^	2016	Netherlands	RCT	0, 12	64	67	50.3±1.1
Wiegant *et al*^22^	2015	Netherlands	RCT	0, 3, 6, 12, 24	67	100	52.6±0.8

The Review Manager (RevMan) version 5 software was used for statistical analysis. Cochrane Q tests and Forest Plots were employed to compare outcomes among KJD, HTO and TKA.

**Fig. 2:**
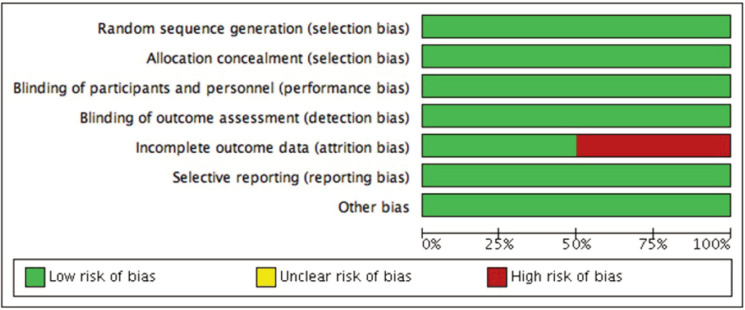
Risk of bias graph: review authors’ judgements about each risk of bias item presented as percentages across all included studies

**Fig. 3:**
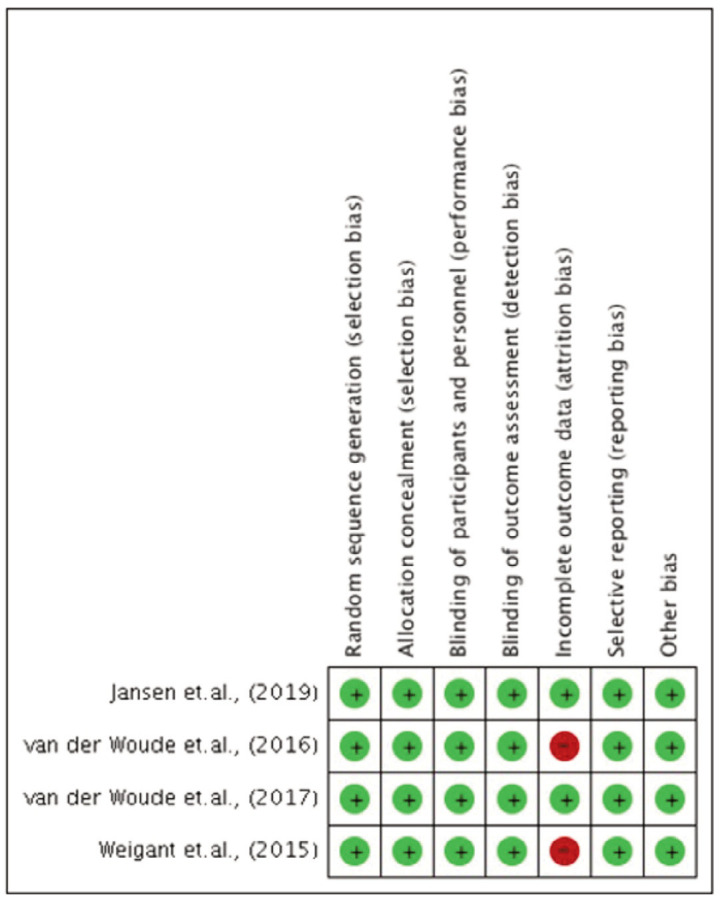
Risk of bias summary and graph: review authors’ judgements about each risk of bias item presented as percentages across all included studies.

Fig. 2 and 3 shows risk of bias graphs illustrate the evaluation of included studies. Studies by Jansen *et al*^19^ and van der Woude *et al*^20,21^ demonstrated low risk for random sequence generation, allocation concealment, blinding, and selective reporting. However, van der Woude *et al*^20,21^ and Wiegant *et al*^22^ had a high risk of bias for incomplete outcome data due to dropouts and attrition.

The researcher declared no conflicts of interest or external funding sources for this study. All data were securely stored on a password-protected hard drive accessible only to the main author and co-proponent. Once the data were analysed, all included studies were permanently deleted.

## Results

This chapter shows the salient findings of the research study. The presentation, interpretation, and analysis of data are shown according to the objectives drawn in the investigation.

**Fig. 4:**
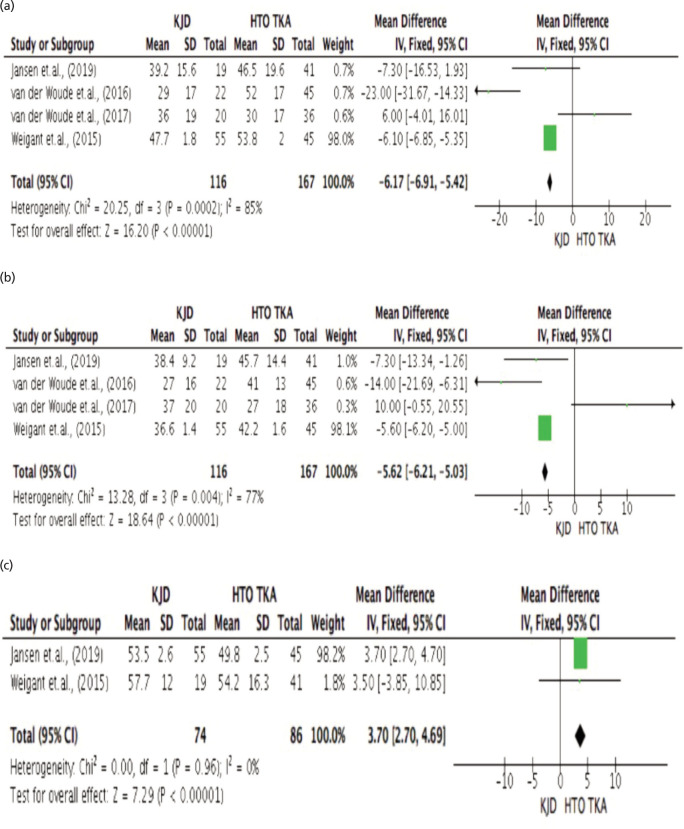
(a) Comparative assessment between Knee Joint Distraction (KJD) and High Tibial Osteotomy with Total Knee Arthroplasty (HTOTKA) in terms of patient related outcome specifically the Western Ontario and McMaster Universities Arthritis Index (WOMAC) score. (b) Comparative assessment between Knee Joint Distraction (KJD) and High Tibia Osteotomy with Total Knee Arthroplasty (HTOTKA) in terms of patient related outcome specifically the Knee Injury and Osteoarthritis Outcome (KOOS) score. (c) Comparative assessment Knee Joint Distraction (KJD) and High Tibial Osteotomy with Total Knee Arthroplasty (HTOTKA) in terms of patient related outcome specifically Intermittent and Constant Pain (ICOAP) Score.

Fig. 4a, presents the comparative assessment between Knee Joint Distraction and High Tibial Osteotomy with Total Knee Arthroplasty in terms of patient related outcome specifically the Western Ontario and McMaster Universities Arthritis Index (WOMAC) score. The results revealed that the highest mean WOMAC score in the KJD group was 47.7±1.8 reported by Wiegant *et al*^22^, similarly, this group of authors also reported the highest WOMAC mean score in the HTO group (53.8±2.0). The results revealed that there was significant difference between KJD and HTOTKA in terms of WOMAC score, with HTO_TKA_ disclosing a not that lower mean WOMAC score (37.98 vs 45.58; p.000 <.05; I^2^=85%). The pooled estimates for mean difference was -6.17 [=6.91 to -5.42].

Fig. 4b, presents the significant difference between Knee Joint Distraction and High Tibial Osteotomy with Total Knee Arthroplasty in terms of patient related outcome specifically the Knee Injury and Osteoarthritis Outcome (KOOS) score. The data showed that the highest mean KOOS score recorded in KJD was 38.4±9.2. This was reported by Jansen *et al*^19^, in which the highest mean KOOS score in the HTO_TKA_ group (45.7±14.4). The results disclosed significant difference between KJD and HTO in terms of KOOS score with HTOTKA disclosing a higher KOOS score over KJD (38.98 vs 34.75; p.000 <.05; I^2^=77%). The pooled estimates for mean difference was -5.62 [-6.21 to -5.03].

Fig. 4c, shows the comparative assessment between Knee Joint Distraction and High Tibial Osteotomy with Total Knee Arthroplasty in terms of patient related outcome specifically the Intermittent and Constant Pain (ICOAP) score. The research of Wiegant *et al*^22^, reported the highest mean ICOAP score for the KJT group (57.7±12). The highest mean score for ICOAP (54.2±16.3) in the HTOTKA group was also reported by Wiegant *et al*^22^. The results revealed that there was significant difference between Knee Joint Distraction and High Tibial Osteotomy with Total Knee Arthroplasty in terms Intermittent and Constant Pain (ICOAP) score. Specifically, KJD scored higher compared to HTOTKA group (55.60 versus 52.00; p.000 <.05; I^2^=0%). The pooled estimates for mean difference was 3.70 [-3.85 to 10.85]).

**Fig. 5:**
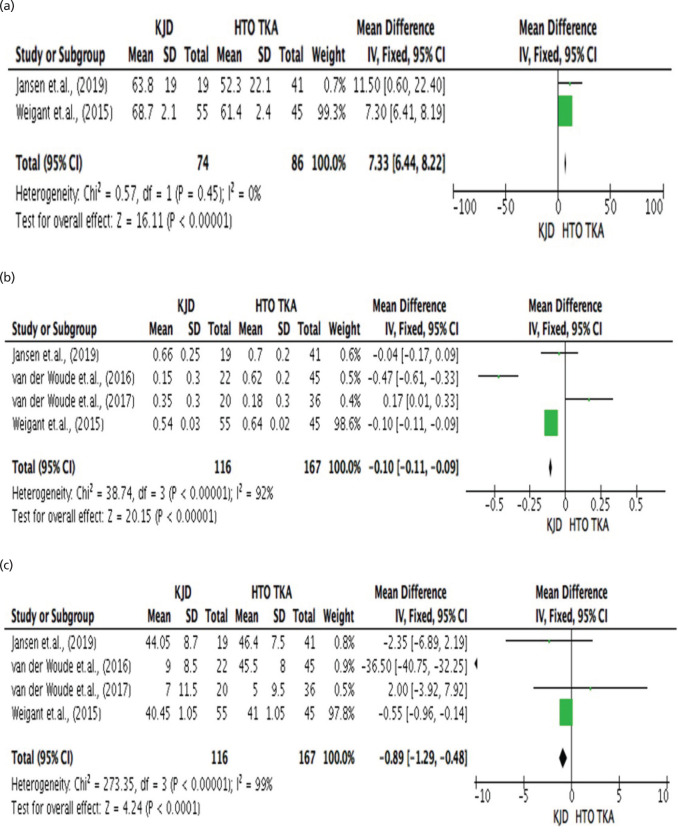
(a) Comparative assessment between Knee Joint Distraction (KJD) and High Tibial Osteotomy with Total Knee Arthroplasty (HTOTKA) in terms of Patient Related Outcome specifically visual analogue scale (VAS) Score. (b) Comparative assessment between Knee Joint Distraction (KJD) and high tibial osteotomy with total knee arthroplasty (HTOTKA) in terms of patient related outcome specifically Euro Quality of life 5D score (EQ5D). (c) Comparative assessment between Knee Joint Distraction (KJD) and High Tibial Osteotomy with Total Knee Arthroplasty (HTOTKA) in terms of patient related outcome specifically the Short Form Health Survey (SF36) score.

Fig. 5a, shows the comparative assessment between Knee Joint Distraction and High Tibial Osteotomy with Total Knee Arthroplasty in terms of patient related outcome specifically Visual Analog Scale (VAS) score. In the study, Wiegant *et al*^22^, reported the highest mean pain score for both the KJD and HTO_TKA_ groups (68.7±2.1 and 61.4±2.4). The results of the study revealed that there was a significant difference between KJD and HTO_TKA_ in terms of patient related outcome specifically their VAS score. The study noted lower mean pain score in the HTO_TKA_ group compared to the KJD group (56.85 vs 66.25; p.000 <.05; I^2^=0%). The results had a pooled estimate for mean difference of 7.33 [6.44 to 8.22]).

Fig. 5b, shows the comparative assessment between Knee Joint Distraction and High Tibial Osteotomy with Total Knee Arthroplasty in terms of patient related outcome specifically the Euro Quality of Life 5D Score (EQ5D). In the study, the highest mean score for EQ5D for KJD group (0.66±0.25) was reported by Jansen *et al*^19^. In the HTO_TKA_ group, Jansen *et al*^19^, reported also the highest mean score of EQ5D (0.7±0.2). In the study, statistical difference between KJDTKA and HTO_TKA_ was observed. The results revealed that HTO_TKA_ scored higher mean EQ5D compared to the patients under the KJDTKA group (0.54 vs. 0.43; p.000 <.05; I_2_=92%). Although the difference in mean score was not markedly wide. The pooled estimates for mean difference was -0.10 [-0.11 to -0.09].

Fig. 5b, presents the significant difference between Knee Joint Distraction and High Tibial Osteotomy with Total Knee Arthroplasty in terms of patient related outcome specifically the Short Form Health Survey (SF36) score. Jansen *et al*^19^, reported the highest mean average for SF36 in patients under the KJD group (44.05±8.7). Similar group of researchers, specifically the team of Jansen *et al*^19^, noted the highest mean score for SF36 in the HTO_TKA_ group (46.4±7.5). The data showed that there was a significant difference between KJD and HTO_TKA_ in terms of the Short Form health survey (SF36). In the study, a higher mean score for SF36 was observed in the HTOTKA group while a lower mean score was noted in the KJD group (34.38 vs 25.13; p.000 <.05; I^2^=99%). The pooled estimates for mean difference was -0.89 [-1.29 to -0.48].

**Fig. 6:**
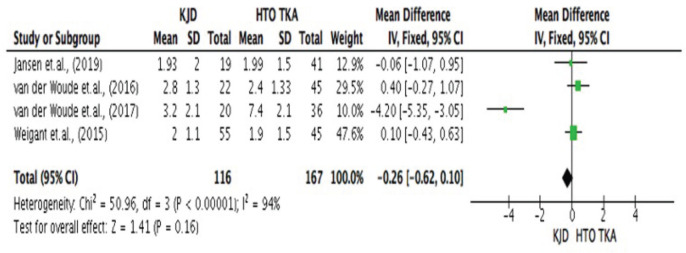
Comparative assessment between Knee Joint Distraction (KJD) and High Tibial Osteotomy with Total Knee Arthroplasty (HTOTKA) in terms of Structural Outcome specifically the Mean Joint Space Width (JSW)

For secondary outcome, the Fig. 6, shows the significant difference between Knee Joint Distraction and High Tibial Osteotomy with Total Knee Arthroplasty in terms of structural outcome specifically the mean Joint Space Width (JSW). In the study, the highest mean joint space width was reported by van der Woude *et al*^20,21^, in the KJD group (3.2±2.1). Similarly, the highest mean joint space width (7.4±2.1) in the HTOTKA group was reported by van der Woude *et al*^20,21^, the results of the study revealed statistical significance between KJD and HTO_TKA_ in terms of mean joint space width (JSW). In the study, a higher mean JSW was observed in patients under the HTO_TKA_ group compared to those placed in the KJD_TKA_ treatment group (3.42 vs 2.48; p.000 <.05), though the mean difference was observed to be not that high. The pooled estimates for mean difference was -0.26 [-0.62 to 0.10].

## Discussion

Knee osteoarthritis (KOA) is one of the most common degenerative joint diseases, particularly affecting elderly individuals. It results in progressive cartilage degradation, leading to pain, loss of joint function, and decreased quality of life. The global prevalence of KOA is estimated at 34%, with a higher incidence observed in women. The pathogenesis of KOA is multifactorial, involving mechanical, genetic, and biochemical factors^23-25^. In advanced stages of KOA, surgical intervention is often necessary to relieve symptoms and restore joint function.

Among the various treatment options for severe KOA, Total Knee Arthroplasty (TKA) remains the standard approach for end-stage disease. However, due to concerns over prosthesis longevity, risk of complications, and the increasing number of younger patients requiring surgery, alternative joint-preserving procedures like Knee Joint Distraction (KJD) and High Tibial Osteotomy (HTO) have gained attention^5-6^. These techniques aim to preserve the native joint and delay or avoid the need for TKA.

In this study, we compared KJD, HTO, and TKA in terms of both patient-reported outcomes and structural improvements in patients with severe KOA. The results provide valuable insights into the comparative effectiveness of these procedures, offering clinicians guidance in selecting appropriate interventions for specific patient populations.

The Western Ontario and McMaster Universities Osteoarthritis Index (WOMAC) is one of the most widely used outcome measures for assessing KOA. Our analysis revealed a significant difference between KJD and TKA (p < 0.05), with TKA showing a lower mean WOMAC score (37.98 vs 45.58). This indicates that patients undergoing TKA experience greater functional improvement and pain relief. Similar findings were reported by Jansen *et al*^19^, who highlighted the significant functional improvements and pain relief achieved with TKA compared to KJD. Additionally, Jansen *et al*^19^ observed comparable short-term functional improvements for both KJD and HTO, though TKA showed an advantage in long-term outcomes.

The Knee Injury and Osteoarthritis Outcome Score (KOOS) demonstrated a similar trend, with TKA patients reporting higher scores (45.7 vs 38.4 for KJD), indicating better functional outcomes. The significant difference (p<0.05) between KJD and TKA is supported by studies such as Jansen *et al*^19^, which showed that TKA led to superior scores across domains such as symptoms, function, and quality of life when compared to both KJD and HTO. Despite this, KJD remains a viable option for patients not yet in need of a full knee replacement.

Pain assessment through the Intermittent and Constant Osteoarthritis Pain (ICOAP) and Visual Analog Scale (VAS) scores revealed higher pain levels among KJD patients compared to those undergoing TKA. While the difference in ICOAP scores between KJD and TKA (55.6 vs. 52.0) was statistically significant (p<0.05), the mean difference was small. However, the VAS score revealed a more substantial pain reduction for TKA patients (56.85 vs 66.25), consistent with findings by Jansen *et al*^19^. These results reinforce the notion that TKA provides superior pain relief compared to KJD.

Health-related quality of life measures, such as the EuroQol 5D (EQ5D) and Short Form Health Survey (SF36), further supported the superiority of TKA. TKA patients reported higher EQ5D (0.54 vs. 0.43) and SF36 (34.38 vs. 25.13) scores, reflecting more substantial improvements in both physical and mental health. Intema *et al*^10^ corroborated these findings, demonstrating that TKA significantly enhances physical and psychosocial well-being, underscoring its effectiveness for advanced KOA.

Structural changes, as measured by Joint Space Width (JSW), showed a significant difference between KJD and TKA. The TKA group demonstrated a higher mean JSW (7.4), suggesting that TKA offers more substantial joint space restoration. This finding is consistent with research by Jansen *et al*^19^, which highlighted the capability of KJD and HTO to improve cartilage quality. However, TKA remains superior in terms of pronounced structural improvements, particularly regarding joint space restoration.

KJD, which involves external fixation to distract the joint and promote cartilage regeneration, showed promising outcomes for joint space restoration and symptom relief. However, pain scores (VAS and ICOAP) remained higher than those observed with TKA, highlighting a limitation in long-term pain relief. As noted in studies by Jansen *et al*^19^ and Intema *et al*^10^, KJD is most suitable for patients aiming to delay TKA, particularly younger individuals with a preference for joint-preserving approaches^8,26^.

HTO, involving the realignment of the knee joint to redistribute weight and relieve pressure on affected areas, also demonstrated benefits in structural improvements and pain reduction. However, its outcomes were not as pronounced as those achieved through TKA. HTO remains a viable option for patients with unicompartmental KOA and is particularly advantageous in younger, more active populations aiming to preserve joint functionality^8,10^.

This study highlights the benefits of both KJD and HTO as joint-preserving procedures for severe KOA. While TKA remains the gold standard for end-stage disease, KJD and HTO provide alternative options that can significantly improve pain relief, function, and joint preservation, particularly in younger patients or those with less advanced disease. Future research should focus on long-term studies assessing the durability of KJD and HTO, including their role in delaying the need for TKA. Investigations into the combination of joint-preserving techniques with adjunctive therapies, such as cartilage repair and stem cell therapy^15,24,27^, may further enhance outcomes for patients with severe KOA^28^.

## Conclusion

Based on the findings of this meta-analysis, the study provides a comprehensive comparison of Knee Joint Distraction (KJD), High Tibial Osteotomy (HTO), and Total Knee Arthroplasty (TKA) in patients with severe knee osteoarthritis. The analysis reveals statistically significant improvements in patient-reported outcomes (such as WOMAC, ICOAP, VAS, EQ5D, and SF36 scores) for both KJD and HTO/TKA. While KJD and HTO/TKA demonstrated comparable outcomes in these parameters, the differences in the scores were not dramatically higher in one group over the other. Specifically, the mean WOMAC, VAS, and EQ5D scores indicated a slight advantage for TKA over KJD, yet the gap was not sufficiently large to definitively favour one treatment modality. Regarding structural outcomes, the mean Joint Space Width (JSW) was found to be statistically significant between KJD and HTO/TKA, with KJD showing potential for cartilage preservation, similar to HTO. These findings support the idea that KJD may alleviate pain and improve joint function effectively, offering a comparable, joint-preserving alternative to HTO and TKA, particularly for younger, active patients where joint replacement may not be the best option due to long-term concerns about prosthesis failure.

Although HTO showed slightly better outcomes in certain patient-related parameters, the differences were not substantial enough to unequivocally favour it over KJD. Therefore, knee joint distraction can be considered a viable alternative to high tibial osteotomy and total knee arthroplasty, especially for patients who wish to avoid or delay the need for joint replacement surgery. Further long-term studies with larger sample sizes and a broader range of outcome measures are needed to solidify the role of KJD in the management of severe knee osteoarthritis.
